# Awareness and knowledge of autism spectrum disorder in Western China: Promoting early identification and intervention

**DOI:** 10.3389/fpsyt.2022.970611

**Published:** 2022-11-10

**Authors:** Hua Wei, Yan Li, Yu Zhang, Jinmei Luo, Shuang Wang, Qiujun Dong, Yuanyuan Tao, Ling Gong, Yang Feng, Mingmei Shi, Zhenghui Cao, Yongfang Liu, Li Chen, Xiao Liu, Ying Dai, Lingling Qu, Zhao Song, Jie Chen, Tingyu Li, Qian Cheng

**Affiliations:** ^1^Department of Child Health Care, Children's Hospital of Chongqing Medical University, National Clinical Research Center for Child Health and Disorders, Chongqing, China; ^2^Ministry of Education Key Laboratory of Child Development and Disorders, Chongqing Key Laboratory of Child Health and Nutrition, Chongqing, China; ^3^Department of Child Healthcare, Changshou District Maternal and Child Care Family Planning Service Center, Chongqing, China; ^4^Department of Children's Rehabilitation, Wushan Maternal and Child Health Hospital, Chongqing, China; ^5^Department of Nutrition, Children's Hospital of Chongqing Medical University, Chongqing, China

**Keywords:** autism spectrum disorder, awareness, early detection, predictors, China

## Abstract

**Purpose:**

Given the increasing prevalence of autism spectrum disorder (ASD) and the public health problems it creates; early identification and interventions are needed to improve the prognosis of ASD. Hence, this study surveyed different groups of people who are likely to have early contact with autistic children to provide an informed basis for early detection and effective diagnosis and interventions.

**Methods:**

Three groups of people were recruited for the study from Changshou District and Wushan County of Chongqing, in Western China: 269 medical workers, 181 educators, and 188 community residents. Their understanding and knowledge of autism was measured using a self-made questionnaire.

**Results:**

The positive finding was that the three groups had a certain level of understanding of autism, but they had some misunderstandings of the core problems, and there were significant differences in the understanding of autism among the three groups. Younger medical workers knew more about autism than older ones did. The ability of educators and community residents to identify autistic symptoms was positively related to their level of education and their experience with autistic children. Television and the internet were the main sources of information about autism for participants.

**Conclusions:**

The medical workers, educators, and community residents in the investigated areas in western China may be able to identify early signs of autism but have an inadequate understanding of autism. In areas far from cities, it is necessary to strengthen the training of medical workers in primary health care to promote autism screening and referral in educational institutions and communities. Using internet technology to provide public education and professional training about autism in remote areas could be a very promising method in Western China.

## Introduction

Autism spectrum disorder (ASD) is a neurodevelopmental disorder characterized by impairments in social communication and restricted and repetitive patterns of behavior, interests, or activities (1). Early identification of ASD and providing services for it is a huge challenge. The prevalence of ASD has increased significantly over the past decade. The World Health Organization (WHO) estimated that 0.76% of the world's children has ASD (2). One in 44 children who are 8 years-old have been estimated to have ASD in the United States (3). Current evidence indicates that interventions to increase the functionality of children with ASD is more effective at young ages and improves long-term prognosis (4). Therefore, raising awareness about autism among people who could identify autism in its early stage is very important (5). Earlier recognition and diagnosis will help parents devise well-constructed and streamlined treatment plans that help reduce stress (6).

Research on ASD was initiated relatively late in China. Previous studies have shown that the prevalence of autism is relatively low in China. Until recently, the Chinese population study identification scheme, which uses standardized cases, reported that the estimated prevalence rate was close to that in the west—i.e., equivalent to 0.7% (7). Some studies indicate that lack of ASD awareness, the low probability of receiving an ASD diagnosis, and stigma may be key factors leading to insufficient recognition of autistic children and major obstacles to treatment and education (8–10). In the past 30 years, the number of studies on autism has gradually increased in China, but research on ASD awareness is relatively rare. A cross-sectional survey found that < 15% of child health care workers in southwest China knew that ASD is a developmental disorder (11). In Southern China, one study (*N* = 471) reported that 83% of preschool teachers inaccurately answered more than half of the questions that assessed knowledge about ASD (12). Another study reported that a higher proportion of Chinese citizens attached stigma to ASD than did citizens in the United States (13). However, there have been few surveys of different populations in the same geographical area, such as medical workers, educators, and community residents in China.

Awareness of autism is relatively high in areas with relatively developed economies in China (e.g., Central and Eastern China), and early screening for ASD has been established in communities in some areas that rely on China's three-level child health care system (14). Chongqing, which is the only municipality directly under the Central Government in western areas of China, is a group of urban areas consisting of a large city (Chongqing Downtown), six regional central cities (with a population of over one million), and 25 other counties. Yet, no studies have investigated the awareness of ASD among different people in this area, and some counties in Chongqing have not yet conducted autism interventions. This study investigated the awareness of autism in different population groups, including medical workers, educators, and community residents, as they are probably the persons who are contacted first and most often by autistic children. The purpose of the present study was to improve understanding of autism among relevant people in this area, to develop a basis for early detection and more effective diagnoses and interventions for ASD.

## Materials and methods

### Study sites and participants

Chongqing is the largest city in western China, with an area of 82,400 square kilometers and a population of more than 30 million. Chongqing is the most representative western city in China. We used stratified random sampling to divide the districts and counties in Chongqing into high and low levels according to their economic level. A district or county was randomly selected from each level to be included in the study (Changshou District and Wushan County). The Changshou District, which is located around the central urban area, is a county with a net population in-flow, and its economic level is at the upper-middle level of Chongqing. Wushan County, which is located at the city boundary in Chongqing, is a county with net population out-flow, and its economic level is at the lower-middle level of Chongqing. The study was designed as a cross-sectional survey that distributed questionnaires to different groups to determine differences in their attitudes toward and knowledge of autism. We used cluster sampling method to conduct a sampling survey on hospitals, educational institutions, and communities under the jurisdiction of local maternal and child health centers. The study was approved by the Ethics Committee of the Children's Hospital of Chongqing Medical University, Chongqing, China. All questionnaires were voluntarily completed after informed consent was provided. The respondents were divided into a medical worker group, an educator group, and a community resident group. The sample size of this study was based on the calculation formula of the cross-sectional study. Relevant research has shown that 57% to 65% of surveyed Chinese citizens (*N* = 1,254) have adequate knowledge of ASD, including its diagnosis, symptoms, etiology, and treatment (13); therefore, this was the estimated value of the expected P in this study: The sample size calculation formula is:


$$N=\frac{Z\alpha^{2}}{d^{2}}\times P\left(1-P\right)$$

*P* = 0.65. Admissible error d = 0.1P, α = 0.05. The sample size required for each region was 206. Considering sampling error and invalid questionnaires, 20% was added to the estimated sample size, so the sample size in each region was at least 247. A total of 643 questionnaires were collected, including 638 qualified questionnaires (Changshou District *N* = 373, Wushan County *N* = 265), for an effective questionnaire rate of 99.2%; invalid data were not included in the statistical analysis. A total of 269 medical workers, 181 educators, and 188 community residents participated in the survey.

### Questionnaire content

The researcher prepared the first draft of the questionnaire, with some questions based on previous studies (15). Then the questionnaire was adjusted based on the Diagnostic and Statistical Manual of Mental Disorders-5th edition (DSM-5) (1) and evidenced-based interventions for ASD (16), as well as the current state of autism interventions in China (17). It was validated by a multidisciplinary team of experts in child developmental behavior, psychology, epidemiology, and primary health care. The questionnaire consisted of three parts and a total of 20 items. The first part collected the basic data and demographic characteristics of the participants, including age, sex, educational level, occupation, and whether participants had experience with autistic children. The second part contained questions about knowledge of ASD, including its severity, description, etiology, and manifestations. The third part asked about awareness of and attitudes toward early detection of autism, treatment institutions, treatment methods, prognosis, and access to information about autism. The criteria for identifying autism manifestations were whether the symptom answers selected by the respondents, which included descriptions of social communication and restricted and repetitive patterns of behavior, interests, or activities; this was defined as “able to identify autism.” If the selected symptoms did not completely include the main symptoms, this was defined as “not completely able to identify autism.” The third category was defined as “unclear.” Other questions were classified and evaluated according to the actual selection of survey items. We invited 20 community residents in Changshou and Wushan to pre-test the questionnaire. The respondents reported whether the questionnaire was easy to understand and in line with local conditions. The questionnaire was adjusted according to their feedback.

### Procedures

The local medical and education departments and relevant community departments at the survey sites uniformly distributed the electronic questionnaire. There was no time limit for completing the questionnaire, which usually took 10–15 min to complete. If participants had any questions, the trained investigators answered them during the survey. Participants did not need to register, and they completed the questionnaire anonymously. There was an informed consent form at the beginning of the questionnaire, and participants could answer questions after they agreed. Investigators scanned the questionnaire's Quick Response (QR) code using mobile WeChat. The WeChat (https://weixin.qq.com) was created by Tencent Technology Company and it is free to download and use. The web-based survey tool Sojump (Changsharan Xing InfoTech, Ltd,) was used to create electronic questionnaires, form the QR code of the questionnaire, and collect data. Sojump (http://www.sojump.com) is a free professional evaluation and polling platform that provides personalized services, including questionnaire design, data collection, and customized reports.

### Statistical analyses

The results were presented as frequencies (%) for categorical data. Chi-square tests were used to analyze differences in categorical variables across the three groups. Multiple regression was performed to explore variables that might predict recognition of autism symptoms. The multiple regression analysis was performed using the stepwise method to screen variables. The inclusion criterion of variables was SLE = 0.15, and the exclusion criterion of variables was SLS = 0.10. The recognition of autism was a dependent variable. The control variables included in the multiple regression model were gender, age, occupation of medical workers, educational level, and experience with autistic children. These variables were assigned the following codes: gender (1 = female and 2 = male); age (1 = < 30 years-old, 2 = 30–40 years-old, and 3 = > 40 years-old); occupation of medical workers (1 = doctor, 2 = nurse, and 3 = medical worker); educational level (1 = High school or less and 2 = Higher education); and experience with autistic children (1 = no, 2 = yes). Tests of significance were two-tailed, and *p* < 0.05 was statistically significant. All statistical analyses were performed with the SAS 9.4 software package for Windows.

## Results

### Sample characteristics

The demographic characteristics of the 638 study participants are presented in Table 1. There was no significant difference in the average age of the three groups. The educational level of the medical workers and educators were significantly higher than the level of community residents (*p* < 0.001). Just over a quarter (26.4%) of medical workers and 28.7% of educators had experience with autistic children, compared to 8% of community residents. A total of 220 medical workers were engaged in clinical work, including 90 doctors and 130 nurses, and 49 were engaged in technical work. Approximately 30% (*n* = 82) of medical workers worked in the Department of Pediatrics, 23% (*n* = 63) worked in the Department of Child Health Care, 7% (*n* = 19) worked in the Department of Rehabilitation, 5% (*n* = 15) worked in the Department of Neurology, 1% (*n* = 3) in the Department of Psychology, and 1 worked in the Department of Psychiatry.

**Table 1:** Characteristics of the three groups (*N* = 638).

**Characteristic**	***n*** **(%)**			***P***
	**Medical workers (*n =* 269)**	**Educators (*n =* 181)**	**Community residents (*n =* 188)**	
**Gender**				
Male	40 (14.9)	4 (2.2)	47 (25.0)	<0.001
Female	229 (85.1)	177 (97.8)	141 (75.0)	
**Age (years)**				
<30 years old	107 (39.8)	65 (35.9)	82 (43.6)	0.283
30–40 years old	96 (35.7)	58 (32.0)	62 (33.0)	
> 40 years old	66 (24.5)	58 (32.0)	44 (23.4)	
**Education level**				
High school or less	14 (5.2)	30 (16.6)	110 (58.5)	<0.001
Higher education	255 (94.8)	151 (83.4)	78 (41.5)	
**Experience with autistic children**				
Yes	71 (26.4)	52 (28.7)	15 (8.0)	<0.001
No	198 (73.6)	129 (71.3)	173 (92.0)	

### Awareness and knowledge of ASD

There were significant differences in knowledge about autism among the three groups (Table 2). Most participants in three groups thought autism is a serious disorder. A total of 45.74% (*n* = 86) of the community residents thought that autism is a rare disorder, whereas 40.33% (*n* = 73) of educators and 34.20% (*n* = 92) of medical workers thought it is rare. The three groups varied widely in their opinions about the intellectual level of autistic children, and most failed to know that autism is a developmental disorder. Only 24.91% (*n* = 67) of medical workers, 24.86% (*n* = 45) of educators, and 14.89% (*n* = 28) of community residents thought autism is a developmental disorder; there was statistically significant difference across groups (*p* = 0.001). Most participants thought autism is a psychological problem. There was a statistically significant difference across groups in their recognition of the main symptoms of autism (*p* < 0.001). The recognition rate was highest in the group of medical workers.

**Table 2:** Awareness and baseline knowledge of ASD among the three groups (*n*, %).

	**Medical workers**	**Educators**	**Community residents**	***P***
**Is autism a serious disorder?**			
Yes	235 (87.36)	137 (75.69)	149 (79.26)	
No	11 (4.09)	29 (16.02)	12 (6.38)	<0.001
Unclear	23 (8.55)	15 (8.29)	27 (14.36)	
**Is autism a rare disorder?**			
Yes	92 (34.20)	73 (40.33)	86 (45.74)	
No	161 (59.85)	91 (50.28)	63 (33.51)	<0.001
Unclear	16 (5.95)	17 (9.39)	39 (20.74)	
**What do you think of the intellectual development of autistic children?**			
Normal	39 (14.50)	33 (18.23)	43 (22.87)	
Higher than normal	20 (7.43)	14 (7.73)	4 (2.13)	<0.001
Lower than normal	19 (7.06)	9 (4.97)	21 (11.17)	
Some are lower and some are normal or high	166 (61.71)	104 (57.46)	83 (44.15)	
Unclear	25 (9.29)	21 (11.60)	37 (19.68)	
**What does autism belong to?**			
Psychological problems	169 (62.83)	105 (58.01)	129 (68.62)	
Developmental disorders	67 (24.91)	45 (24.86)	28 (14.89)	0.001
Psychiatric disorders	21 (7.81)	6 (3.31)	7 (3.72)	
It's not a **disorder**, it's a personality problem	5 (1.86)	10 (5.52)	10 (5.32)	
Unclear	7 (2.60)	15 (8.29)	14 (7.45)	
**Identification of core symptoms of autism** ^**a**^			
Able to identify	184 (68.40)	50 (27.62)	39 (20.74)	
Not completely able to identify	78 (29.00)	124 (68.51)	139 (73.94)	0.001
Unclear	7 (2.60)	7 (3.87)	10 (5.32)	

a Questions on the symptoms of autism are: Children with autism are indifferent to their surroundings, often play alone and exhibit social withdrawal; Children with autism often don't respond to people calling their names; Children with autism may not be able to speak when they should be able to. Many children with autism have communication difficulties; Many autistic children have repetitive stereotyped behaviors or narrow interests, such as repeatedly rotating objects or slapping their arms.

### Awareness of the causes of autism

**Figure 1:**
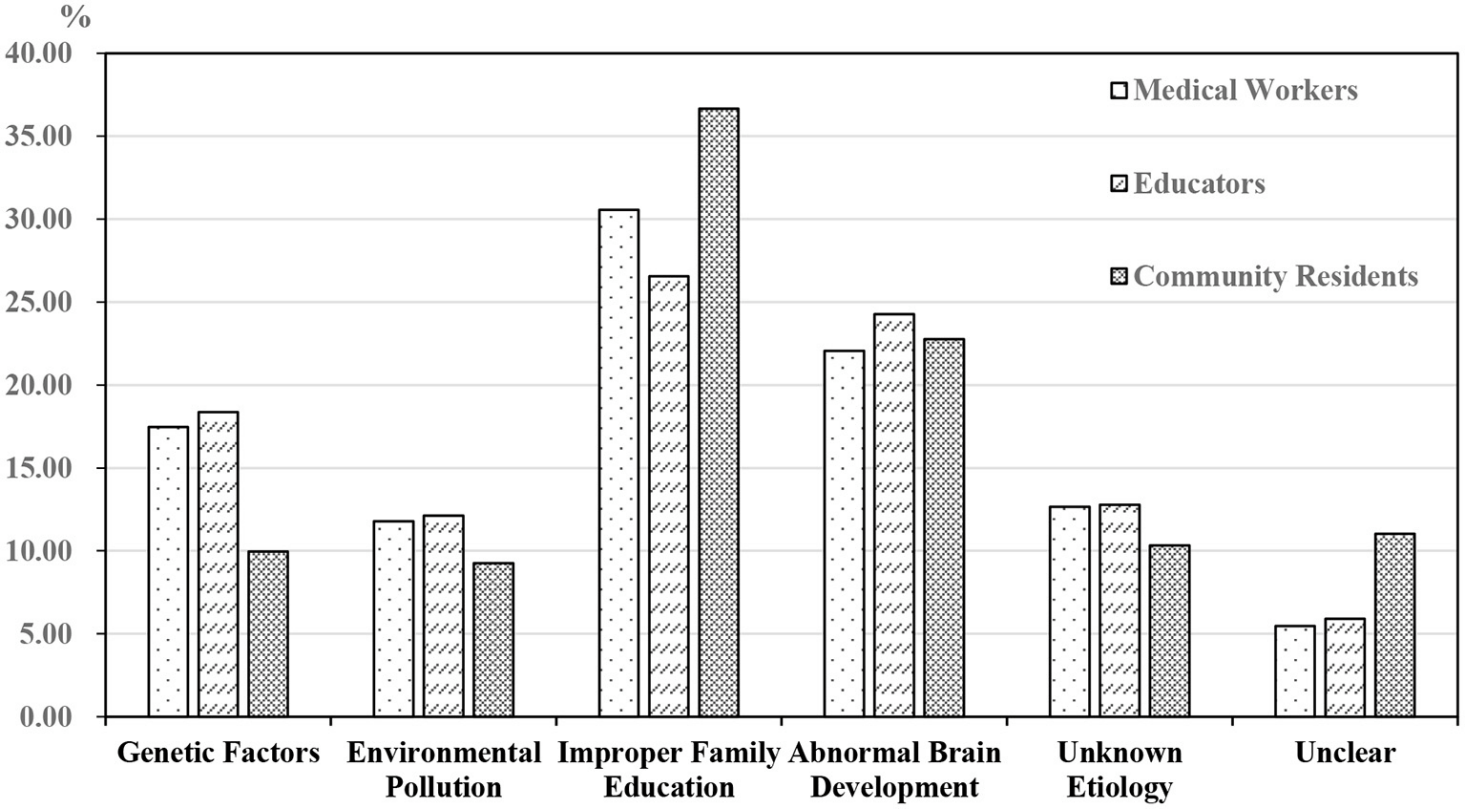
Awareness of the causes of autism. The question is what do you think is the possible cause of autism? The answer is multiple choice, but if the choice is “unclear,” the respondents can't choose other options.

More than a quarter of participants in each group thought autism was caused by “improper family education” (Figure 1): this included 30.57% (*n* = 140) of medical workers, 36.65% (*n* = 103) of community residents, and 26.56% (*n* = 81) of educators. The second most frequently selected cause in each group (22% to 25%) was “abnormal brain development.”

### Awareness of early detection and choice of department to visit for help

In response to the question, “What do you think if a child can't communicate until the age of 2, or has little eye contact with others?” most of the people in each group (91.71% to 98.51%) thought that they should go to the hospital for professional help (see Table 3). When asked what hospital department they should go to for help, most chose primary health care, followed closely by the psychology department of a hospital.

**Table 3:** Awareness of early identify and seeking help (*n*, %).

	**Medical workers**	**Educators**	**Community residents**	***P***
**What do you think if a child can't communicate until the age of 2 or has little eye contact with others?**				
Go to the hospital for professional help	265 (98.51)	166 (91.71)	177 (94.15)	
Wait and see how the child develops	2 (0.74)	5 (2.76)	5 (2.66)	0.01
Unclear	2 (0.74)	10 (5.52)	6 (3.19)	
**If you choose to communicate with doctors, which department will you choose?**				
Primary health care	215 (36.07)	121 (31.51)	126 (32.56)	
Psychology Department	190 (31.88)	121 (31.51)	120 (31.01)	0.10
Psychiatry Department	55 (9.23)	37 (9.64)	46 (11.89)	
Neurology Department	52 (8.72)	30 (7.81)	32 (8.27)	
Rehabilitation Department	36 (6.04)	27 (7.03)	19 (4.91)	
Pediatrics Department	45 (7.55)	36 (9.38)	31 (8.01)	
Unclear	3 (0.50)	12 (3.13)	13 (3.36)	

### Awareness and knowledge of interventions for ASD

The vast majority of participants in each group agreed that autism requires treatment (Table 4). Nearly half (48.70%, *n* = 131) of medical workers said the most effective intervention is special education training, whereas 46.41% (*n* = 84) of educators and 55.85% (*n* = 105) of community residents said psychological counseling is best; there was a statistically significant difference across the groups' choices of treatment methods (*p* < 0.001). More than 9 out of 10 (92.57%, *n* = 249) medical workers thought that autism requires long-term interventions, whereas 77.90% (*n* = 141) of educators and 77.60% (*n* = 146) of community residents thought long-term treatments are needed for autistic children. When asked, “Where can autistic children get interventions?,” the most common responses were hospitals, rehabilitation institutions, and psychological clinics. More than 9 in 10 (91.71%, *n* = 166) educators and 89.59% (*n* = 241) of medical workers said autistic children should receive special education services in school, whereas only 79.26% (*n* = 149) of community residents thought they should; the difference across groups was statistically significant (*p* < 0.001).

**Table 4:** Awareness of interventions for ASD (*n*, %).

	**Medical workers**	**Educators**	**Community residents**	***P***
**Does autism need treatment?**				
Yes	265 (98.51)	172 (95.03)	181 (96.28)	
No	0 (0.00)	6 (3.31)	2 (1.06)	0.09
Unclear	4 (1.49)	3 (1.66)	5 (2.66)	
**What is the most effective treatment for autism?**				
Special education training	131 (48.70)	73 (40.33)	57 (30.32)	
Behavioral therapy	15 (5.58)	7 (3.87)	6 (3.19)	
Psychological counseling	114 (42.38)	84 (46.41)	105 (55.85)	<0.001
Medical treatment	4 (1.49)	2 (1.10)	5 (2.66)	
Unclear	5 (1.86)	15 (8.29)	15 (7.98)	
**Do autistic children need long-term interventions?**				
Yes	249 (92.57)	141 (77.90)	146 (77.66)	
No	5 (1.86)	14 (7.73)	9 (4.79)	<0.001
Unclear	15 (5.58)	26 (14.36)	33 (17.55)	
**Access to intervention services for child with autism** ^**a**^				
Hospital	199 (32.25)	96 (23.76)	122 (30.65)	
Rehabilitation institutions	159 (25.77)	107 (26.49)	78 (19.60)	
Psychological clinic	139 (22.53)	95 (23.51)	103 (25.88)	<0.001
Treatment at home	82 (13.29)	60 (14.85)	59 (14.82)	
Public/private educational institutions	36 (5.83)	29 (7.18)	20 (5.03)	
Unclear	2 (0.32)	17 (4.21)	16 (4.02)	
**Is it important for autistic children to get special education services in school?**				
Yes	241 (89.59)	166 (91.71)	149 (79.26)	
No	18 (6.69)	6 (3.31)	14 (7.45)	<0.001
Unclear	10 (3.72)	9 (4.97)	25 (13.30)	

a The answer is multiple choice, but if the choice is “unclear” the respondents can't choose other options.

### Awareness of the prognosis of autism and factors that influence autism

More than half (52.79%, *n* = 142) of medical workers and 47.87% (*n* = 90) of community residents thought autism can be completely improved by early intervention, compared to 36.46% (*n* = 66) of educators (Table 5). The percent of educators who thought autism could be partially improved by early intervention was significantly higher than that of the other two groups (*p* < 0.001) ~ 25–30% of participants in each group said early detection and treatment can improve the prognosis of autism.

**Table 5:** Awareness of the prognosis of ASD and influencing factors.

	**Medical workers**	**Educators**	**Community residents**	***P***
**What is the improvement of ASD symptoms by early intervention**				
Completely improves	142 (52.79)	66 (36.46)	90 (47.87)	
Partially improves	118 (43.87)	94 (51.93)	76 (40.43)	<0.001
Unclear	9 (3.35)	21 (11.60)	22 (11.70)	
**What factors affect the prognosis of autism?** ^**a**^				
Early detection and treatment	262 (25.49)	166 (29.96)	139 (29.83)	
IQ level	90 (8.75)	45 (8.12)	37 (7.94)	<0.001
Language ability	134 (13.04)	79 (14.26)	81 (17.38)	
The severity of autism symptoms	177 (17.22)	91 (16.43)	74 (15.88)	
Comorbidity	158 (15.37)	51 (9.21)	15 (3.22)	
Family participation	203 (19.75)	118 (21.30)	106 (22.75)	
Unclear	4 (0.39)	4 (0.72)	14 (3.00)	

a The answer is multiple choice, but if the choice is “unclear,” the respondents can't choose other options.

### Access to ASD information

Television and the internet were the main sources of information about autism for participants, with hospitals being a far less common third source (Table 6). Medical workers were more likely to obtain information about autism from newspapers and books than the other two groups were. All three groups were more likely to choose parent training, publicity about autism, and early screening as being helpful for autistic children and their families than other community services were, such as peer support or welfare funds (there was no significant difference across the three groups, *p* = 0.49).

**Table 6:** Access to ASD information.

	**Medical workers**	**Educators**	**Community residents**	***P***
**How do you get information about autism?**				
Television	195 (21.57)	116 (24.12)	122 (28.18)	
Internet	221 (24.45)	142 (29.52)	134 (30.95)	
Newspapers	102 (11.28)	43 (8.94)	30 (6.93)	
Books	115 (12.72)	48 (9.98)	29 (6.70)	<0.001
Community	100 (11.06)	32 (6.65)	36 (8.31)	
Hospital	157 (17.37)	80 (16.63)	57 (13.16)	
Other	10 (1.11)	16 (3.33)	17 (3.93)	
Unclear	4 (0.44)	4 (0.83)	8 (1.85)	
**What community services can help autistic children and families?**				
Parent training	219 (20.22)	120 (19.26)	124 (20.70)	
Community autism screening	201 (18.56)	108 (17.34)	98 (16.36)	
Community intervention training	169 (15.60)	104 (16.69)	97 (16.19)	0.49
Peer support (Integrated Education)	170 (15.70)	87 (13.96)	81 (13.52)	
Community publicity related knowledge	227 (21.14)	148 (23.76)	140 (23.37)	
Welfare Fund	95 (8.77)	56 (8.99)	59 (9.85)	

### Predictors of awareness and knowledge of autistic symptoms

The results of the multiple regression on variables predicting the ability to correctly identifying symptoms of autism are shown in Table 7. Gender, age, occupation, educational level, and experience with autistic children were the possible predictors. Gender, age, and occupation were negatively associated with medical workers' ability to identify autistic symptoms (*p* < 0.05). The results showed that young female medical workers, especially doctors, had relatively better ability to identify autism, compared to other medical workers. The educational level of the teacher group was positively related to their ability to recognize autism (*p* < 0.05), wherein teachers with higher education levels had a greater ability to identify autism. Educational level was also positively related to the ability to identify autism in the community residents group (*p* < 0.001); that is, community residents with higher educational levels had a greater ability to identify autism. No significant correlation was found between gender and the ability to identify autism. The ability to identify autism was better (*p* < 0.05) for the educator and community residents groups, and people who had experience with autistic children.

**Table 7:** Predictors that the three groups recognized autism symptoms.

**Dependent variable**	**Independent variable**	**Regression coefficient (**β**)**		***P***	**R2/R2adj**
		**Parameter estimate**	**Standardized estimate**		
Recognition in medical workers	Gender	−0.17135	0.08094	<0.05	0.0684/ 0.0507
	Age	−0.00477	0.00230	<0.05	
	Occupation	−0.07139	0.03230	<0.05	
	Experience with autistic children	0.29852	0.18257	0.103	
Recognition in educators	Educational level	0.14081	0.06666	<0.05	0.0833/0.0677
	Experience with autistic children	0.20696	0.08454	<0.05	
Recognition in community residents	Gender	0.12252	0.07864	0.121	0.1309/0.1167
	Educational level	0.17457	0.04403	<0.001	
	Experience with autistic children	0.23956	0.10128	<0.05	

## Discussion

Early detection and diagnosis are important prerequisites for obtaining a good therapeutic effect for ASD (18). The public's lack of understanding and knowledge of autism may lead to delays in its diagnosis and interventions for it, especially among those people who can observe very early manifestations of autism (19). ASD in China differs substantially from ASD in the West, in terms of its prevalence estimates and the educational opportunities and life outcomes of the people who have it. The lack of awareness of ASD in China could be a key factor underlying these disparities. It also makes it difficult to have early identification of and interventions for autism, especially in areas with insufficient autism services in China.

In our research, the two study areas differed in their geographical locations, economic development, and population flow. Some studies have shown that economies and cultures may affect the public's knowledge, stigmatization, and identification of autism (20–22). The flow of population can also affect the ideology of a region. An in-flow of population can increase communication among residents and bring different ideas. There are many migrant workers in Wushan County, and most of the child caregivers are elderly people at home, so the information to them is relatively limited. At the beginning of the survey design, we speculated that there would be differences in the awareness of autism between the two regions. However, initial analyses found no significant difference in the awareness of ASD between the two areas. This may be because public education and/or professional development/training on autism have not been conducted in either region in the past.

This paper further analyzes and discusses differences in understanding autism among three different populations. It also provides a basis for relevant government departments to conduct community health professional training and health education, and to conduct targeted publicity and education, or professional knowledge training, to promote the early detection and diagnosis of autistic children as soon as possible.

Overall, most people in the three groups knew of autism and had some simple knowledge about autism, which is the “good news” of our study. Medical workers had a better understanding of autism-related information and recognition of autistic symptoms. However, some misunderstandings about ASD were found by this survey. Most people in all three groups thought autism was uncommon. This was especially true in the educator and community resident groups. Many studies have also reported that variations in ASD incidence rates are related to the ability to identify and diagnose autism in different populations (23, 24). A Japanese survey of parents' and teachers' estimates of the prevalence of ASD found the estimates of both groups were consistently very low (25). Most participants in our study thought that autism is a psychological problem and that improper family education is the main cause of autism. Other studies have shown that medical workers have insufficient understanding of autism (26, 27). Educators in schools and childcare centers are a vital resource because they are likely to be able to identify developmental delays early (28). Some studies have shown that educators had a baseline knowledge of ASD, but their knowledge was inadequate or lacked specific details (29, 30). Community residents are also important members of the public. Parents of children with autism have reported that lack of community knowledge about autism leads to feelings of stigmatization and stigma toward their children (31, 32). Community residents' insufficient understanding or misunderstanding of autism may cause parents to feel ashamed that their children have autism, think that their children will improve when they are older, or be unwilling to take their children to a hospital as soon as possible. The limited understanding or an incorrect understanding of ASD is likely to have an adverse impact on the early identification of autism in the three groups we studied.

Early identification is key to diagnosing ASD. At present, the doctors who can screen and diagnose autism in China are mainly developmental-behavioral pediatricians, child health doctors, psychologists, and psychiatrists (17). However, there are few developmental-behavioral pediatricians, psychologists, or psychiatrists in districts and counties far from cities in China, so primary health care workers undertake most of the work of medical treatment and referral (33). Most of the people in this study chose to go to primary health care and psychology departments for a diagnosis. In China, some pediatric psychologists also undertake the work of developmental-behavioral pediatricians. In the two areas where we conducted our investigation, autism screening was started in the local child-health hospital or maternal and child care family planning service center in 2017, but they lacked professionals and diagnostic tests for autism. Therefore, general knowledge and training about autism is very important in these areas. The doctors in relevant departments need to disseminate correct information about autism to the public, especially, to educators, parents, and other community residents.

More than 90% of the participants in each group recognized that autism requires an intervention, and medical workers and educators agreed that autism requires long-term training and recognized the importance of special education in schools, which is positive awareness. Effective treatment for children with ASD involves an interdisciplinary approach. It includes a combination of educational interventions, behavioral therapies, speech-language therapy, occupational/physical therapy, and medical treatment (e.g., psychopharmacology). Evidence-based research shows that effective intervention methods include Application Behavior Analysis (ABA), Naturalistic Teaching Strategies, and Pivotal Response Treatment (16). In China, intervention for children with ASD is a relatively new field. Interventions for children with ASD in China are mainly performed by professionals in hospitals, rehabilitation institutions, and special schools (34). Autism intervention training has not been conducted in the areas we investigated. Children with ASD can only receive physical therapy in the local child health department or go to larger cities for ASD interventions. Moreover, it is relatively difficult to conduct special education or integrated education in ordinary schools because community residents will worry whether it will affect the learning of children with normal development. Early school transitions are challenging experiences for children with special needs, such as ASD, and their families (35). Therefore, it is very important to promote cooperation between families and schools. Most of the participants in our three groups chose psychological counseling as an effective treatment for ASD. This reflects a misunderstanding of ASD and its treatment. The three groups were optimistic about the effects of interventions, but the public needs to recognize that the effects of interventions on some autistic children are not always good. Some studies have shown that understanding the etiology and prognosis of autism may be more helpful for parents to support their autistic children (36), which suggests that we should teach parents more current scientific knowledge about autism as this can guide them to choose proper treatment methods and build their confidence in rehabilitation.

Medical workers in our survey were more likely to identify the core symptoms of autism than educators and community residents were, which may be because medical workers can obtain more relevant knowledge. Television and the internet were the main ways of obtaining knowledge of autism in our study, but medical workers can acquire more information from hospitals, communities, newspapers, and books than the other two groups can. In China, the maternal and child health service network includes maternal and child health institutions and primary medical and health institutions, which provide health services for women and children. Primary health care workers receive relevant medical training on a regular basis. Many regions have conducted community autism screening and referral (14). Our survey showed that some medical workers have work experience in child healthcare departments, rehabilitation departments, and other relevant departments. They may have more opportunities to acquire autism-related knowledge. This suggests we should choose the best ways to publicize autism-related knowledge to different target groups. Child health care workers can improve local autism awareness through networks, community publicity, campus publicity and other means. The younger female medical workers were, the more they knew about autism in our study. A report by Mohammad also showed that younger physicians were more knowledgeable about potential causes of ASD than older physicians were (37). In districts and counties far from cities, younger medical workers are more likely to accept new knowledge and new things through the Internet and receive relevant training. Our results showed that improving the educational level of residents and teachers is probably helpful to improve understanding of autism, as shown in another study (12). With respect to their expectations of community services, the three groups chose more opportunities for community training, screening, intervention training, and peer support than financial subsidies. This suggests that with the improvement in the national economic level, people need more education and training opportunities for autistic children.

For underserved areas, public education about autism is needed and/or the professional development/training for medical workers, educators, and communities needs to be improved. Given the differences in people's understanding of autism, targeted training for different groups should be conducted, which should be presented through a variety of methods. Remote technology based on in the internet has the potential to provide timely and low-cost alternatives to expand access to specialist services to people in remote locations, especially considering the global Corona Virus Disease 2019 (COVID-19) situation (38, 39). Sohl and Mazurek's team used the Extension for Community Healthcare Outcomes (ECHO) method to ASD in the United States (40). An expert team provides mentorship through case-based discussion and recommendations *via* multipoint video conferencing. The results showed that this model builds knowledge and fosters expertise among PCPs, enabling them to provide family-centered, best-practice care, while reducing health care inequities. In many remote areas of China, autism-related services are lacking or insufficient. However, there are maternal and child health care systems. Therefore, based on China's three-level maternal and child health care system, we plan to conduct further public education and professional development/training on autism in the two surveyed areas of Chongqing in the future. We will explore the mode of combining remote training with on-site guidance in the hope of improving autism services in Western China further.

## Conclusions

Our survey on awareness of information about autism included a wide range of content in Western China. Overall, most people knew of autism and had basic knowledge about autism, but some misunderstandings existed. It is necessary to strengthen the training of medical workers in primary health care to promote autism screening and referrals in educational institutions and communities. Using information technology to provide training on autism related knowledge for people in remote areas is also a very promising method.

## Limitations of the study

This study has some limitations. First, the study was based on a self-report questionnaire, which may be subject to reporting bias. Second, in our questionnaire, there was no section about the stigma of autism, which limits our further discussion on the impact of knowledge level and culture on public awareness of ASD. In addition, this was not a wider range of population-based study, the result only represented the situation of the selected region in western China. Further multicenter, large-scale studies are needed to investigate awareness of and knowledge about ASD in China better.

## Data availability statement

The raw data supporting the conclusions of this article will be made available by the authors, without undue reservation.

## Ethics statement

The study was approved by the Ethics Committee of the Children's Hospital of Chongqing Medical University (Ref No. 201932), Chongqing, China. All questionnaires were voluntarily completed after informed consent was provided.

## Author contributions

HW designed the study and wrote the initial draft of the manuscript. YLi, YZ, JL, SW, QD, and YT trained investigators. LG, YF, MS, ZC, LC, XL, YD, LQ, and ZS contributed to data collection. YLiu was responsible of statistical studies. QC, JC, and TL supervised the study process, reviewed, and revised the manuscript. All authors approved the final manuscript as submitted and are responsible for all aspects of the work.

## Funding

This work was supported by the Chongqing Science and Technology Bureau Research Project (Technological Innovation and Application Development in Chongqing) (cstc2019jscx-msxmX0217), the Chongqing Medical Scientific Research Project (Joint Project of Chongqing Health Commission, and Science and Technology Bureau) (2019MSXM037), the National Nature Science Foundation of China (82002399), and the Key Scientific and Technological Projects of Guangdong Province (Grant Number 2018B030335001) and Guangzhou City (Grant Number 202007030002).

## Conflict of interest

The authors declare that the research was conducted in the absence of any commercial or financial relationships that could be construed as a potential conflict of interest.

## Publisher's note

All claims expressed in this article are solely those of the authors and do not necessarily represent those of their affiliated organizations, or those of the publisher, the editors and the reviewers. Any product that may be evaluated in this article, or claim that may be made by its manufacturer, is not guaranteed or endorsed by the publisher.
